# A model to prioritize access to elective surgery on the basis of clinical urgency and waiting time

**DOI:** 10.1186/1472-6963-9-1

**Published:** 2009-01-01

**Authors:** Roberto Valente, Angela Testi, Elena Tanfani, Marco Fato, Ivan Porro, Maurizio Santo, Gregorio Santori, Giancarlo Torre, Gianluca Ansaldo

**Affiliations:** 1 Health Management Unit, S. Martino University Hospital, L.go R. Benzi 10, 16132 Genoa, Italy; 2 Department of Economy and Quantitative Method, University of Genoa, Via Vivaldi 5, 16126 Genoa, Italy; 3 Department of Communication, Computer and System Sciences, University of Genoa, Via all'Opera Pia 13, 16145 Genoa, Italy; 4 Department of Transplantation, S. Martino University Hospital, L.go R. Benzi 10, 16132 Genoa, Italy; 5 General Surgery Clinic, Department of Surgery, S. Martino University Hospital, L.go R. Benzi 10, 16132 Genoa, Italy

## Abstract

**Background:**

Prioritization of waiting lists for elective surgery represents a major issue in public systems in view of the fact that patients often suffer from consequences of long waiting times. In addition, administrative and standardized data on waiting lists are generally lacking in Italy, where no detailed national reports are available. This is true although since 2002 the National Government has defined implicit Urgency-Related Groups (URGs) associated with Maximum Time Before Treatment (MTBT), similar to the Australian classification. The aim of this paper is to propose a model to manage waiting lists and prioritize admissions to elective surgery.

**Methods:**

In 2001, the Italian Ministry of Health funded the Surgical Waiting List Info System (SWALIS) project, with the aim of experimenting solutions for managing elective surgery waiting lists. The project was split into two phases. In the first project phase, ten surgical units in the largest hospital of the Liguria Region were involved in the design of a pre-admission process model. The model was embedded in a Web based software, adopting Italian URGs with minor modifications. The SWALIS pre-admission process was based on the following steps: 1) urgency assessment into URGs; 2) correspondent assignment of a pre-set MTBT; 3) real time prioritization of every referral on the list, according to urgency and waiting time. In the second project phase a prospective descriptive study was performed, when a single general surgery unit was selected as the deployment and test bed, managing all registrations from March 2004 to March 2007 (1809 ordinary and 597 day cases). From August 2005, once the SWALIS model had been modified, waiting lists were monitored and analyzed, measuring the impact of the model by a set of performance indexes (average waiting time, length of the waiting list) and Appropriate Performance Index (API).

**Results:**

The SWALIS pre-admission model was used for all registrations in the test period, fully covering the case mix of the patients referred to surgery. The software produced real time data and advanced parameters, providing patients and users useful tools to manage waiting lists and to schedule hospital admissions with ease and efficiency. The model protected patients from horizontal and vertical inequities, while positive changes in API were observed in the latest period, meaning that more patients were treated within their MTBT.

**Conclusion:**

The SWALIS model achieves the purpose of providing useful data to monitor waiting lists appropriately. It allows homogeneous and standardized prioritization, enhancing transparency, efficiency and equity. Due to its applicability, it might represent a pragmatic approach towards surgical waiting lists, useful in both clinical practice and strategic resource management.

## Background

### Waiting lists and prioritization

Waiting lists for elective surgery (WLES) are problematic for public healthcare systems, because patients often experience long waiting times with a negative impact on health and quality of life [1-4]. WLES represent dynamical sets where behavior is unpredictable and policy interventions are difficult to assess [5]. Since the 1960s, research has shifted in the field of prioritization with the aim of ensuring prompt access for patients most in need. Although several models were proposed [5], they were based on different principles without great international agreement. Different tools were developed for elective surgery either based on implicit semi quantitative or explicit quantitative criteria [6-8]. The choice between these criteria is an ongoing point of discussion: implicit criteria are more easily applicable but generally lack definition, whereas explicit criteria are not unequivocally agreed upon and are often perceived as too inflexible [9-12]. In the State of Victoria (Australia), implicit categories of clinical urgency were identified and applied to all elective surgical registrations [13,14]. The Australian Government adopted the classification, delivering WLES national reports [15]. An application of real time systems to surgical waiting lists was described in 1999 by Davis and Johnson, who developed a computerized model to get a "Patient's Eligibility Quotient" starting from a "Patient's Initial Quotient"[16]. In 2002, following the Australian experience, the Italian Government adopted implicit criteria to prioritize admission to elective surgery on the basis of four clinical Urgency-Related Groups (URGs) [17]. Each URG was associated with a period of time within which admission should be provided ("Maximum Time Before Treatment", MTBT). Nevertheless, the application of URGs' proved difficult and Italian patients are generally admitted on a first-in first-out basis, taking into account broad and subjective views of urgency.

### Italian health care context

At present, in the Italian public health care system patients are referred to surgeons by their General Practitioner. Patients are free to choose the surgical unit anywhere in the Public System, and upon making their specialist appointment are charged a "ticket" fee depending on the Region or possible exemption. Waiting times for the visit may vary from only a few days to several months, depending on speciality and local facilities. Surgical visits take place within the public hospital, by salary paid surgeons at which time patients are put on elective waiting list, at no extra charge. At any time patients are free to undergo private consultation and treatment, choosing where and by whom they are operated on.

### Aim of the study

The aim of this paper is to propose and discuss a model to monitor and manage waiting lists and prioritize admissions to elective surgery on the basis of clinical urgency and waiting time.

## Methods

### SWALIS research project

In 2001, the Italian Ministry of Health provided 200,000 Euro to fund the Surgical Waiting List Info System (SWALIS) project [18,19], with the aim to improve the management of WLES by information technologies. The SWALIS project was developed with resources and skills in economics (Department of Economy and Quantitative Method, DIEM – University of Genoa), and technology-telematics (Department of Communication, Computer and System Sciences, DIST – University of Genoa). Clinical skills and environment were provided by the S. Martino University Hospital in Genoa, a public general hospital that serves a regional area with about 1,500,000 inhabitants and performs over 30,000 surgical admissions at a total cost of approximately 138,000,000 Euro each year. It is one of the largest in Northern Italy, with a total of 1,618 beds, 674 of which are for surgery. Project participants and the Regional Health Administration department formed a multidisciplinary steering committee. The SWALIS Project started in January 2002 following an organizational improvement model used as a roadmap for initiating, planning, and implementing improvement actions (IDEALSM Model) [20]. The SWALIS Project was split into a two-year model development phase (January 2002 – February 2004), followed by a three-year experimentation phase (March 2004 – March 2007).

### Phase 1: model development

#### Local current practice evaluation: preliminary survey

In the first phase of the project (model development), the steering committee performed an informal preliminary survey among the local staff of each surgical unit in the S. Martino University Hospital. A retrospective audit was then run gathering information from previous available data, searching for waiting list composition and length, management process and clinical prioritization model. Some of the most common elective surgical procedures were accessed after a long wait, in some cases lasting more than one year. The composition of waiting lists was difficult to measure because of the lack of comparable data, partly due to the contemporary internal administrative re-organization within the hospital. Only from 2005 was some comparable information available. However, waiting list related reports were considered inadequate because waiting start and end points were often undetectable, both because the database had a heterogeneous structure and because much of the data was missing.

#### Definitions of urgency and priority

According to the Australian definition, we refer to "urgency" as the pressingness of the clinical condition and the promptness of the necessary treatment, regardless of the patient being on a waiting list [15]. The level of urgency results from a clinical assessment through the use of selected criteria. Clinical urgency can be associated to a time interval in which treatment is considered desirable and/or appropriate, both for clinical and contextual reasons. Alternatively, we refer to "priority" as the current need for treatment of each of the patients at an index time, once they are placed on a waiting list. Similar to the Davis and Johnson's definition of Patient Eligibility Quotient [16], we consider priority to be the measure of the increasing clinical need as time goes by, as a result of the application of an algorithm. Priority is then objectively obtained as a score, determining the order of patients on the list.

In line with this distinction of the two definitions, patients gain different eligibility ("priority") during their waiting period on account of the original clinical condition ("urgency").

#### Model definition and system requirements

Before implementing the SWALIS informative system prototype, a modeling study was performed by our group in order to simulate the impact of a prioritization-scoring-algorithm of this kind [21]. The steering committee selected ten representative surgical units within the Hospital and formed a scientific committee of referee surgeons and head nurses from each unit, including general surgery, colorectal surgery, vascular surgery, cardiac surgery, thoracic surgery, ophthalmic surgery, orthopedic surgery, and gynecologic surgery. The scientific committee set system requirements by following a simplified methodology to reach an agreement on the prioritization model and on the functionalities of the new software environment. Referees produced delivery at the end of each meeting, at the beginning of which they were allowed free interaction in the presence of a facilitator [22]. Output of each session was subjected to a multiple revision process and finally delivered to system developers.

#### Clinical urgency classification

The scientific committee adopted Italian URGs with further minor definitions, based on two implicit semi quantitative criteria: 1) the presence of fast disease progression; 2) the grade of pain, dysfunction or disability. Each URG is associated with a MTBT, as shown in Table 1.

**Table 1:** SWALIS modified Italian Government urgency related groups (URGs)

**URG**	**Clinical assessment**	**MTBT**
A1	Evident fast progression of disease affecting outcome by delay (SWALIS)	8 days
A2	Potential fast progression of disease affecting outcome by delay	30 days
B	Severe pain and/or dysfunction and/or disability, but no fast progression of disease affecting outcome by delay	60 days
C	Mild pain and/or dysfunction and/or disability, but no fast progression of disease affecting outcome by delay	180 days
D	No pain, dysfunction and disability and no fast progression of disease affecting outcome by delay	360 days

Italian Government definition of four urgency-related groups (URG) based on the presence of the fast progression of disease (A) and on the grading of pain, dysfunction and disability (B, C, D). Each URG is associated with the respective Maximum Time Before Treatment (MTBT) as indicated in the right column. The SWALIS scientific committee adopted URGs' minor modification, dividing URG A (presence of fast progression of disease affecting outcome by delay, MTBT 30 days) into A1 and A2, by the further definition in case the disease progression was evident (8 days) or potential (30 days), respectively.

#### SWALIS pre-admission model

Every patient was registered with basic information (evident/suspected diagnosis, expected surgical procedure, URG, date/time) and underwent the same three-step pre-admission process.

##### 1. Urgency assessment at registration

The clinical condition of the patient was evaluated by a surgeon on duty within the surgical unit at the first outpatient visit. Patients were registered on the waiting list in case the necessity of treatment was considered either sure or potential. Registrations were date and time stamped. Urgency was assessed according to the URGs' classification.

##### 2. Assignment of the correspondent MTBT

The MTBT was automatically assigned, respecting the pre-set correspondence to the selected URG as shown in Table 1.

##### 3. Prioritization

URGs were associated with an urgency coefficient, representing the speed at which the clinical need is assumed to increase along with the passing of time. For each URG *v*, the urgency coefficient (*γ*_*v*_) was stated by the ratio between the MTBT of the least urgent URG (i.e. URG D) and the MTBT of the corresponding URG. A time-based algorithm was computed in real time, sorting the waiting list via a dynamic priority function. For each patient the priority score at time *t*, *P*(*t*), was obtained by the linear product of the waiting time at time *t*, (*t*-*t*_0_) where *t*_0 _is the registration time, and the respective urgency coefficient (*γ*_*v*_), such that:

*P*(*t*) = (*t*-*t*_0_) * (*γ*_*v*_)

Following this equation, patients proceeded in the list at different speeds according to their urgency, gaining different priority scores, given the same waiting time. Note that P(t) is expressed in days weighted for urgency coefficient, which is Need-Adjusted-Waiting-Days (NAWDs), standardizing waiting times among different URGs [21].

Patients who were subjected to hospital postponements were still prioritized following the same rule.

#### Informative system prototype

**Figure 1:**
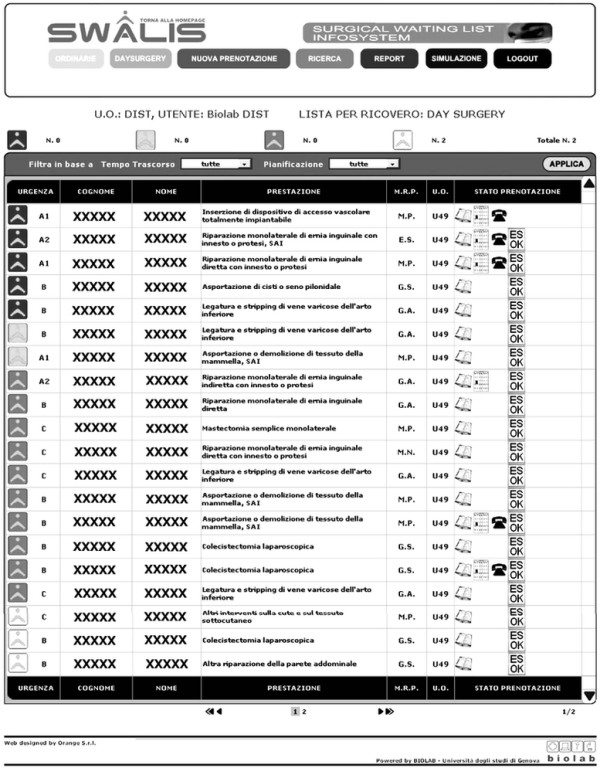
**SWALIS application: waiting list screenshot**. Screen capture of SWALIS system. Software environment was built with a main waiting list display, showing all registrations together with pre-admission summary information in a single recordset ordered by descending priority score.

A system prototype was developed by using open source technologies [23]. The software ordered all registrations in a single priority-sorted list in real time and suggested admissions for patients with the highest priority score, including a short term waiting list forecast (one to four weeks). Priority was displayed to users as a percentage of the MTBT of each patient in the list, so that nurses and doctors had a complete picture in a single screenshot without further operation (Figure 1).

### Phase 2: model experimentation

In the second three-year project phase (model experimentation) the model was deployed and its effects were observed. The hospital's ethic committee disapproved the start of any randomized or control study owing to legal reasons while no retrospective comparison was possible because of the lack of local comparable data prior to the SWALIS model's deployment. A descriptive prospective study was then designed and performed, monitoring the experimentation and measuring the impact of the model by a trend analysis of time series under its homogeneous application.

A single general surgery unit was selected within the ten surgical units as a pilot test bed for deployment and on-field application. At the beginning of phase 2, the model was subjected to a fine-tuning period, from March 2004 to July 2005, when URGs were assigned to all registrations. This was followed by a monitoring period, from August 2005 to March 2007, when the full model was applied in the clinical practice as the standard pre-admission process and waiting lists were measured together with the performance of the prioritization.

Surgeons explained to their patients the URG assignment, the expected MTBT and the prioritization algorithm. Waiting list managers (day-time and head nurses) looked after the process and planned admissions using the SWALIS software. URGs distribution was collected from different categories such as type of admission, presence of neoplasm, International Classification of Diseases (ICD) general coding, and duration of surgery.

### Experimental context control

The project steering committee devised control surveys, collecting information every three months to monitor the experimentation, the involvement of users, their training and feedback, system prototype faults, and waiting list performance. The general status of the experimentation was updated including information about the involvement of the surgical units and the input of new registrations. The continuous system control included the prioritization module and the standard application of the SWALIS admission rule.

Most database parameters were controlled weekly together with the integrity, completeness and update of the waiting list data. Waiting list back office performance indexes were monitored on a monthly basis as soon as they became available.

Surgeons' clinical referees actively collected feedback by their colleagues and by the surgical unit's waiting list managers. Difficulties which arose during data entry, viewing and updating the system, were monitored as well as those in URGs and ICD assessment. Users were also asked about the perceived usefulness of the model. All information was organized into periodical synoptic reports and discussed during the steering committee's quarterly meetings.

### Managing URG assessment subjectivity

The SWALIS project adopted the Italian URGs because of their National value and considered them as rules to be applied focusing on the experimentation of the entire model rather than on the clinical urgency criteria *per se*. Assessment subjectivity was not investigated with proper tools (i.e. inter-rater or intra-rater reliability) in this study; however, other experiences described a relevant subjectivity in the use of similar implicit urgency criteria [24]. With this in mind, meetings with surgeons were organized to reduce this risk, to share the judgment criteria of common clinical conditions and to audit registrations.

Round table meetings took place within the pilot unit every three months. All nine surgeons and the two waiting list managers on staff participated. The clinical referee led the meeting, while the waiting list managers took notes and presented the audit material. Each meeting was divided into two parts.

The first part was semi-structured as a broad inspective internal audit lasting 45 to 50 minutes, during which time reports about registrations of the previous three months were shown. Each of the following points was presented and discussed until surgeons reached a final agreement.

1. Broad analysis of URGs assessment in more common (ICD based) clinical cases.

2. URGs assessment for rare diagnosis and treatments.

3. Single registrations with URG deviating from the more common distribution.

4. Single registrations with reported difficult assessment.

5. Comparison of URG assessments among surgeons.

The second part of the meeting was an unstructured 15 minute open discussion about any relevant problems.

### Measuring waiting lists and prioritization performance

The effects of SWALIS model application were preliminarily observed during the fine-tuning period, early in project phase 2 [25]. Once the SWALIS pre-admission process had been amended and reached consistent standard use (August 2005), waiting lists underwent a monitoring period until March 2007, evaluating their performance by a trend analysis of time series. The impact of the SWALIS model on waiting lists was measured using both retrospective and cross-sectional methods by a new set of indexes (Table 2), in order to quantify waiting list performance [26]. It was followed by the use of the indexes in Table 2 between URGs, including mean waiting times and Appropriate Performance Index (API), as the percentage of patients belonging to a given URG treated within their MTBT. Waiting time was calculated as the retrospective time waited before patient admission, and as the cross-sectional time already waited by patients still on the list on an index day. In a separate and more in-depth paper by some of the authors NAWDS were used to measure the relative clinical need and the performance of waiting lists in terms of equity, according to Culyer's distinction between horizontal and vertical equity [27,28]. Alternatively, in the present study equity attainment was set as the prioritization goal, i.e. that every patient could be admitted according to his/her URG.

**Table 2:** Indexes for monitoring waiting list performance

**Indexes**	**Aggregation level**	**Retrospective**	**Cross-sectional**
**WAITING TIME**	- For each URG	Waiting time of patients who received treatment during a given period *T*	Waiting time of patients currently on the list at an index day
	- All classes		
	
	- For each URG	Waiting time of patients who received treatment during a given period *T*, weighted with the priority score	Waiting time of patients currently on the list at an index day, weighed with the priority score
	- All classes		

**WAITING LIST LENGTH**	- For each URG	Average number of patients on waiting list during a given period *T*	Number of patients currently on the list at an index day
	- All classes		

**APPROPRIATE PERFORMANCE INDEX (API)**	- For each URG	Percentage of patients receiving treatment within their respective MTBT in a given period of time *T*	Percentage of patients currently on the list at an index day having waiting time inferior to their respective MTBT

### Statistical analysis

Results are expressed as mean +/- standard deviation (SD), median, and 95% confidence interval (CI). Graphics and statistical analyses were performed using R software/environment, an open source project that is distributed under the GNU  General Public License (version 3, 29 June 2007; Copyright 2007 Free Software Foundation, Inc.). Sources and binaries for R software can be obtained via Comprehensive R Archive Network . At the time of writing this, R-2.7.1 was available [29].

## Results

### Experimentation descriptive analysis

During the entire three-year model experimentation phase, from March 2004 to March 2007, 45 surgeons and 22 nurses were involved. The SWALIS database was queried and (N = 113) errors were deleted prior to final analysis. During the monitoring period, from August 2005 to March 2007, the system prototype service rate remained constant, waiting lists were fully updated and 100% of admissions in the pilot unit were planned using the SWALIS algorithm. Table 3 presents the descriptive analysis of 2406 consequent registrations, including the overall population of patients admitted and dropped off the waiting list. As the table shows, the pre-admission model was applied to a relevant heterogeneity of clinical conditions. Age resulted to be distributed into a wide range (from paediatric to elderly patients), while the sex variable had a moderate prevalence of female patients, due to breast surgery candidates. URGs were assigned to all patients waiting for ordinary and day case surgery, and whether the complexity of the operation was major, medium or minor (based on the duration of the expected surgery).

The SWALIS model was routinely utilized for several diagnoses and surgical procedures, belonging to most of the general categories (classified by ICD coding), and in the presence (or absence) of benign or malignant neoplasms.

**Table 3:** Overall SWALIS patient population

	**Urgency-related Groups (URGs)**					
	**A1 (N = 192, 7.9%)**	**A2 (N = 897, 37.3%)**	**B (N = 857, 36.6%)**	**C (N = 425, 17.7%)**	**D (N = 35, 1.4%)**	**Total (N = 2406, 100%)**
**Admission**						

Day surgery	57	183	121	221	15	597 (24.8%)
Ordinary	135	714	736	204	20	1,809 (75.2%)

**Neoplasms**						

Malignancy	99	270	18	3	-	390 (16.2%)
Benign or uncertain	15	117	64	11	-	207 (8.6%)
Non neoplasms	78	510	775	411	35	1,809 (75.2%)

**Surgical procedures**						

Abdominal wall and hernias	11	70	90	153	3	327 (13.6%)
Breast	12	130	18	6	-	166 (6.9%)
Chest	5	3	2	-	-	10 (0.4%)
Digestive visceral	46	93	56	11	5	211 (8.8%)
Endocrine	25	349	515	155	12	1,056 (43.9%)
Gynecologic	1	8	13	2	-	24 (0.9%)
Hepatobileopancreatic	18	47	107	9	2	183 (7.6%)
Spleen and lymphatics	15	100	2	1	-	118 (4.9%)
Skin	35	28	15	25	2	105 (4.4%)
Urology and andrology	4	12	9	5	-	30 (1.2%)
Vascular	4	33	20	55	11	123 (5.1%)
Other	16	24	10	3	-	53 (2.2%)

**AGE**	61.6 ± 17.5	56.9 ± 17.1	56.6 ± 15.4	55.1 ± 14.6	59.6 ± 16	56.9 ± 16.2
(median; 95% CI)	(65; from 59.1 to 64.1)	(59; from 55.7 to 58)	(59; from 55.5 to 57.6)	(57; from 53.7 to 56.5)	(64; from 54.1 to 65.1)	(59; from 56.2 to 57.5)

**SEX**						

F	108	612	574	210	25	1,529 (63.5%)
M	84	285	283	215	10	877 (36.4%)

**Expected operating time**						

Undefined procedure	14	195	84	128	5	426 (17.7%)
< 1 h	52	128	145	63	9	397 (16.5%)
Between 1 h and 3 h	118	551	610	229	21	1,529 (63.5%)
> 3 h	8	23	18	5	-	54 (2.2%)

CI: Confidence interval. SWALIS database was queried: table shows frequency analysis of distribution of the overall population among different URGs.

### Use reports

No relevant conflict emerged from either users or patients. The system provided functionalities to schedule admissions and appointments, establishing how patients' priority would change in relation to the expected waiting time.

During control surveys, waiting list managers reported the SWALIS system to be a useful tool to reduce postponements due to more efficient planning. They also described the perception of an increase of safety, due to the possibility to check for exceeding waits at a glance rather than going through the paper registry.

Surgeons reported some initial apprehension about structuring their clinical judgment and entering a more auditable area. They also expressed some concern about the competition of their respective patients due to the risk of subjectivity in URGs assessment as described in Victoria [24]. Nonetheless, they reported that the periodical meetings (see Methods section) allowed them to deal with the problem and to share a common approach. As experimentation proceeded, surgeons' wariness reduced progressively as they were able to promptly attain information about their patients' waiting time. On several occasions they reported feeling comfortable under the protection of a more accountable system in case their patients were subjected to hospital initiated delays and admission postponements.

### Availability of waiting list data: monitoring waiting lists

**Figure 2:**
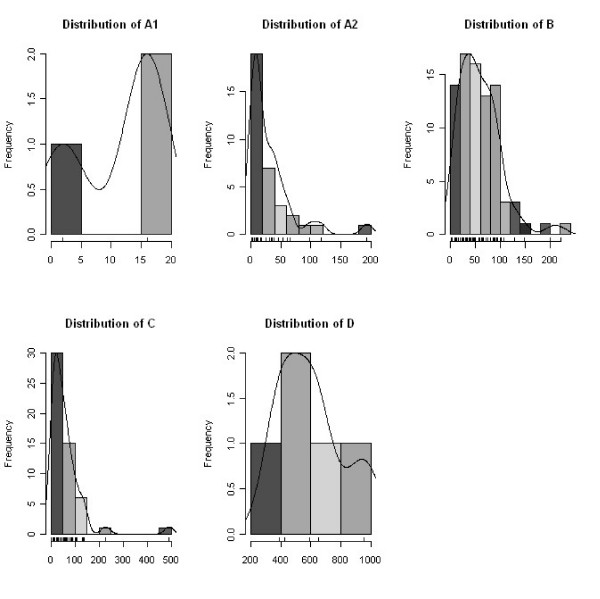
**SWALIS waiting times distribution within URGs (cross – sectional)**. Histograms show the cross sectional frequency distribution of patients present in the list at an index day within each URG (A1, A2, B, C and D). Each bar represents the count of patients (frequency) grouped by periods of waited days on scaled X-axis. URGs classification is shown in Table 1.

**Figure 3:**
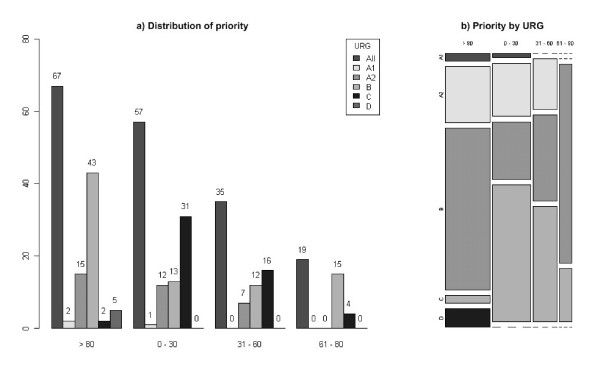
**Overall SWALIS waiting lists priority distribution (cross – sectional)**. Cross sectional frequency distribution of priority among patients present in the list at an index day within each URG (A1, A2, B, C and D). Priority is shown as the integer percentage value of the respective MTBT. The frequency of patients for each URG, grouped by priority range (> 80, 0–30, 31–60, 61–80) is displayed as a raw count (a) and as a relative count (b). URGs classification is shown in Table 1.

**Figure 4:**
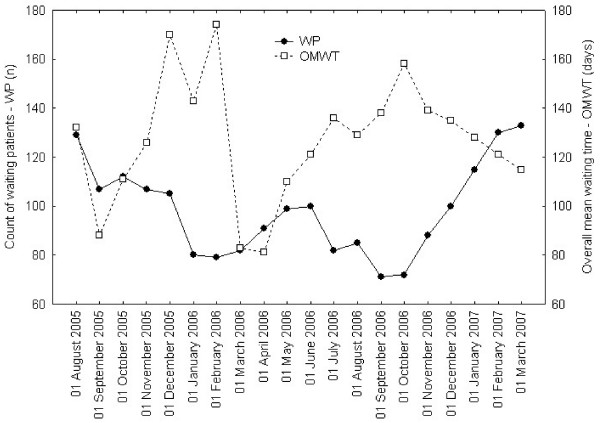
**Cross sectional Waiting patients and overall mean waiting time**. Lines show monthly values at an index day of length of the list (count of Waiting Patients-WP) and Overall Mean Waiting Time (OMWT).

**Figure 5:**
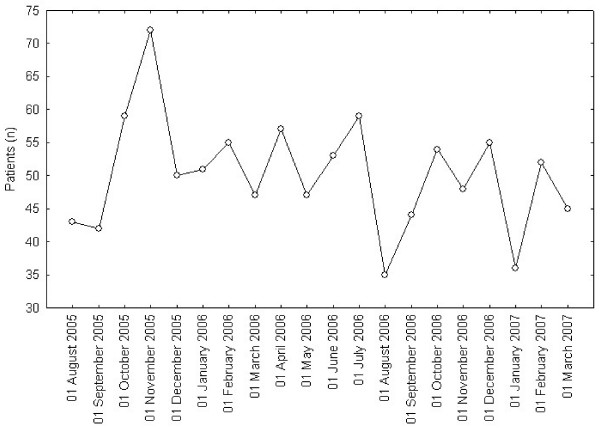
**Retrospective admitted patients**. Lines show monthly values (count) of admitted patients at an index day, in the previous 30 days. During SWALIS experimentation service rate was set as constant.

**Figure 6:**
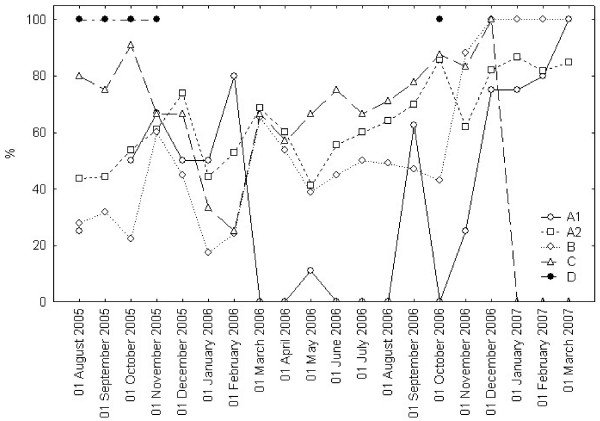
**Appropriate Performance Index (API)**. Lines show monthly values of Appropriate Performance Index (API) of admitted patients at index days, in the previous 30 days, within each URG (A1, A2, B, C and D). API is defined in Table 2. URGs classification is shown in Table 1.

The system prototype provided the rich set of waiting list data and detailed performance parameters described in table 2. Informative reports (days attended minimums, maximums, means) were automatically generated. As shown in figures 2 and 3 by cross-sectional view, the informative system allowed the generation of advanced datasets and runtime graphic displays. The frequency distribution of the waiting patients could be represented within each URG (Figure 2) or in an overall mosaic (Figure 3), bringing a comprehensible visualization of waiting list population. Similar views could be compared at different time-series end point (Figures 4 to 6): clinical users, managers and patients could be informed in real time about the trends of the waiting list and about the current expected waiting times.

### Effects of SWALIS prioritization on waiting list performance

Table 4 reports monthly mean waiting times and overall mean and median waiting times through the test period.

**Table 4:** Cross-sectional and retrospective mean waiting times

	**Cross-sectional mean waiting time (days)**					**Retrospective mean waiting time (days)**				
**Index day**	**A1**	**A2**	**B**	**C**	**D**	**A1**	**A2**	**B**	**C**	**D**
**01/08/2005**	9	46	58	115	461	8	66	72	115	189
**01/09/2005**	7	42	71	114	414	-	80	76	135	185
**01/10/2005**	-	37	49	114	444	7	63	88	149	172
**01/11/2005**	13	28	45	113	429	7	42	77	169	184
**01/12/2005**	-	27	52	144	457	7	38	67	188	-
**01/01/2006**	7	31	54	110	485	8	60	86	212	-
**01/02/2006**	-	17	51	128	520	6	33	81	220	-
**01/03/2006**	4	19	52	74	484	10	25	55	179	-
**01/04/2006**	10	15	51	38	312	12	25	52	169	-
**01/05/2006**	-	18	38	46	340	14	29	68	159	-
**01/06/2006**	-	18	42	57	368	15	28	62	143	-
**01/07/2006**	-	25	38	81	400	13	30	64	195	-
**01/08/2006**	8	26	42	89	419	12	33	61	175	-
**01/09/2006**	11	24	57	101	467	6	35	57	157	-
**01/10/2006**	-	22	27	102	462	11	29	73	132	227
**01/11/2006**	1	22	31	103	490	9	28	68	130	-
**01/12/2006**	5	28	44	100	400	4	25	65	130	-
**01/01/2007**	7	33	48	71	353	8	37	68	142	-
**01/02/2007**	-	35	47	79	381	6	32	71	176	-
**01/03/2007**	5	40	54	93	309	3	38	82	162	-

**Mean ± SD**	7.2 ± 3.3	27.6 ± 8.9	48.5 ± 9.9	93.6 ± 27.0	419.7 ± 61.1	8.7 ± 3.3	38.8 ± 15.7	69.6 ± 10	161.8 ± 28.3	191.4 ± 20.9

**Median**	7	26	48	100	424	8	33	68	160	185

**95% CI**										
From	5.17	23.5	42.9	80.9	391.2	7.1	31.4	65.0	149.0	165.5
To	9.33	31.8	52.2	106.3	448.3	10.3	46.1	74.3	175.1	217.3

SD: Standard deviation. CI: Confidence interval. Mean waiting times were measured in days as cross sectional indexes, calculated among waiting patients at an index day (cross-sectional indexes), and in admitted patients (retrospective indexes).

We demonstrated no trend in length of the list or in the overall mean waiting time, as neither parameter had the tendency to change, though variable in time (Figure 4). Analogous results were observed in patient admission rate (Figure 5), given the service (beds and operation room availability) maintained constant during the whole period.

Data in table 4 show that, with the exception of patients in D category, the overall cross-sectional mean and median waiting times (columns on the left) are inferior to those of the correspondent retrospective ones (columns on the right), i.e. cross-sectional A1 < retrospective A1, cross-sectional A2 < retrospective A2, cross-sectional B < retrospective A1, and cross-sectional C < retrospective C. The overall mean and median waiting times of each URG showed increasing values with the increase of their MTBT, i.e. A1 < A2 < B < C < D, both in cross sectional and retrospective measurements.

As Figure 6 shows, the API of each URG during the test period maintained a variable behavior. No definite change as a trend was demonstrated, although lines in the graph show a tendency to converge to 100% in API at latest time-points.

## Discussion

### Much of WLES behaviour is still unknown

WLES management is a critical issue, since the demand for surgery often overwhelms supply and public systems have limited resources. The application of effective policies is still a rising concern, given no definite standard is available to measure and handle the problem. The matter is then far from being comprehended and its intrinsic complexity has been recognized for more than two decades [30]. Likely as a consequence, detailed information about WLES is difficult to gather in most industrialized countries both from white and gray Internet literature [31]. With very few exceptions, it is hard to know how many patients are currently waiting (or have waited) for specific diagnoses or surgical procedures. Regarding the situation in Italy, recent communications from the Ministry of Health reported scarce homogeneity and availability of waiting list data, promoting innovation by the use of information technologies [32]. Our local context did not represent an exception to the Italian background, since waiting list dimensions and composition at the start of the project were substantially undefined in our experimental area.

### A tool to handle the problem

Given the obscurity of the matter, the SWALIS model was designed with the intention of obtaining transparent data to gain a more in depth understanding of WLES, enhancing prioritization as a leveraging instrument through the use of information technologies. Feasibility and accessibility emerged early on as qualities necessary for the new model, since its application was expected to cause substantial organizational change. The model was then designed incorporating clinical urgency assessment and real time prioritization sequentially. Such a multi-module structure was chosen in order to include the whole pre-admission period, so as to adopt different clinical urgency criteria and to manage any elective surgical procedure. The system was built to provide data and prioritization in a single software environment, becoming a useful tool in the real practice. As a result, the entire demand for surgery was processed consistently, including a very heterogeneous set of clinical conditions and surgical procedures. The integration of adequate indexes within the model allowed the measure of WLES performance, obtaining the necessary information to better understand their composition and trends.

### Effects in practice

The display of patients' respective priority (Figure 1) allowed easy and coherent scheduling. The screenshot view of the waiting list together with the safety warnings allowed nurses and surgeons to detect when patients were at risk of exceeding their MTBT. The waiting list was checked by the waiting list manager following the priority order every time admissions were planned, i.e. at least twice a week. In addition, each surgeon usually controlled the entire list with particular attention to his/her waiting patients, logging on to the system separately and more often. As a consequence of this intense monitoring, waiting lists were kept active and clean from "ghost" patients (patients that have died or gone elsewhere for treatment) preventing evident inequities.

Admissions and surgical resources could be planned quickly by the use of an intelligible forecast, visualizing how many patients – and which of them – were to be admitted in the near future (i.e. in two, three or four weeks). It was easier to keep them in really elective conditions reducing the amount of time they waited. This in turn reduced the need for unexpected bed and operative room occupation for sudden clinical deterioration, often resulting in complicated surgery and prolonged postoperative periods as a consequence of excessive waiting times [3]. Furthermore, on the whole, better planning could reduce postponements by reducing sudden changes in admission schedule.

The availability of waiting time data, either as waiting time distribution (Figures 2 and 3) or mean waiting times (Figure 4), provided reliable and updated estimations of admission dates in real time. This information allowed a more efficient pre-admission path to be planned, avoiding redundancies and unexpected situations (i.e. in planning instrumental and clinical examinations, in the pre-surgery management of anticoagulant drugs suspension or overlap, etc.). By using waiting times indexes, waiting list trends could be analysed in depth and forecast. This allowed for action planning such as sharing reports and organizing meetings among surgeons and waiting list managers in order to solve emerging problems.

The availability of data regarding waiting list composition and dimensions (Figures 2, 3 and 4) allowed managers to plan the allocation of surgical resources and operative room time to each surgeon and to the surgical unit within the department, modulating supply on the base of the measured demand.

Additionally, the standard application of the model to all registrations allowed the SWALIS system to serve as the proper environment to manage the clinical added information (i.e. noting patients' clinical or private necessities). The software allowed relevant synoptic information to be retrieved (i.e. selections of patients, performance indicators, etc.) and to be delivered to respective users, such as surgeons or waiting list and health managers.

### Urgency classification

Urgency assessment is a crucial topic since it represents the main milestone of the entire prioritization process. Given the absence of great international agreement on the subject, it may represent a weak point of our study, since we have not investigated it with proper tools. With this limitation, we started from the application of the Italian implicit urgency criteria because of their statement at a national level. While the rater reliability of the Italian criteria might be further assessed and/or more objectively defined, in our experience they proved to be easily applicable to the entire pool of patients, allowing both coherent data collection and patient prioritization. In order to maintain this adaptability, our model has implied the formal separation of the three steps (urgency assessment, MTBT classification and prioritization), allowing the possible introduction of different clinical criteria if necessary.

### The surgeon's role in the clinical assessment

Urgency assessment was taken into great consideration, since surgeons' clinical judgment was required to be both free and objective. As described in other experiences, a certain amount of guesswork to elevate patients' URG most likely occurred, even though the phenomenon was discouraged and *ad hoc *measures were taken to prevent it from happening [33]. In our experience, contrasts and heterogeneity in clinical evaluations were reduced to an acceptable level through the periodical audit meetings, when surgeons could compare and discuss their assessment, increasing the homogeneity of their criteria. Interestingly, there was never a major conflict of opinions between surgeons while unanimous agreement was always eventually reached. Furthermore, the surgeons' general opinion was that the URG assessment was progressively becoming simpler and more familiar, allowing the necessary individual freedom.

### The patients' point of view

Patients were not asked directly about their experience under SWALIS experimentation, neither in interviews nor by questionnaires, and the impact of the model on patients might be further investigated. With this limitation, during control surveys surgeons and nurses were asked to report issues that emerged at the time of outpatient registration on the list, during the waiting period and at the time of admission.

They described no relevant problem in explaining the criteria of their urgency assessment and reported that patients generally acknowledged that waiting time should not hold the same value for them all but be based on the grade of their illness. On those occasions, patients accepted that waiting time could weigh proportionally to their respective URG and no complaints for being overtaken by more urgent patients were ever reported. We interpreted this finding as mostly due to the national statement of the URGs classification, since an understanding of the prioritization's consistency led to an increase in its level of acceptance [34]. The positive feedback we registered might have been biased by several factors but it was perhaps influenced by the simplicity of the SWALIS model algorithm, and by the patients' perception of a strong Service's engagement in respecting their MTBT.

Regarding the patients' acceptability of waiting periods, it is known that they often suffer from consequences of extensive waiting periods, and that many of them may have different opinions of waiting times [3,4,34]. They should therefore be informed about their expected waiting time and when their admission is most likely to occur, since they often have a better perception of their waiting period the sooner they are given this information [4]. In the SWALIS experience, due to the availability of waiting times in real time, patients could be informed about their expected wait as early as at the time of registration.

### Effects on waiting lists: treating each patient at the right moment

Assuming that free access does not automatically imply immediate healthcare delivery, our model was designed to treat patients at the right moment, satisfying their respective need. The SWALIS model adopted the waiting time as the unique non-clinical criterion, not only admitting patients by classifying them into URGs (and within their MTBT), but also by scheduling admissions using a progressive scoring system [21].

Rather than in waiting list length, the effects of the SWALIS prioritization process are evident within the waiting list composition in real time, since the prioritization algorithm determines a change of their internal sorting continuously, so that every registration will reach the top of the list by its priority score (Figure 1). This clear waiting list behavior allowed the selection of the patients to be admitted first at a glance, simply by looking at the top positions in the screenshot.

As expected, the application of the SWALIS model caused no evident effects in terms of reduction or increase of the overall waiting list length (demand side policy), because resources (beds and operating room availability) was set as constant. Reducing list consistency would require increasing service rate (supply side policy) and the variability shown in Figure 4 depends on variability of arrival rate and of service rate (i.e. admitted patients).

In this study we observed no significant difference in the time series. Even so, by the physiological functioning of the SWALIS model, waiting list observation should result in API moving close to the 100% for all different URGs. We consider this point to need further investigation but this general progressive tendency appears in the latest monthly data in Figure 6.

As some of the authors published separately, the application of the SWALIS model brings an increase in efficiency as well as equity [27]. According to the results of this study, data shown in Table 4 reveal that patients were protected by horizontal and vertical inequities.

Horizontal inequities were avoided because the algorithm calculates priority proportionally to waiting time. With the exception of the few patients in category D (N = 35, 1.4%), this assumption is supported by the comparison of the overall mean and median waiting times of the respective URG, where overall cross sectional waiting times are inferior to the correspondent retrospective ones. This data give evidence to the fact that patients were admitted only after those in the same urgency category that had been waiting longer.

Vertical inequities were avoided by computing the priority on the basis of clinical urgency. The overall waiting times of each URG showed increasing values with the increase of their MTBT, meaning that the more urgent patients were not waiting as long as those in less urgent categories and were admitted earlier.

Table 4 shows some discrepancies in single measurements at Index days in columns A1 and D, where cross-sectional mean waiting times were occasionally higher than retrospective ones, suggesting that a significant part of them were kept on the list, not being admitted before those with shorter waits. Regarding patients in URG A1 (N = 192; 7.9%), this evidence was due to the difficulties in completing the necessary pre admission diagnosis within the short MTBT (eight days). Those patients in fact often required unplanned complex evaluations before admission even while being kept under strict observation by waiting list managers. The small dimension of the A1 subset of patients often caused the single mean values to change more likely as a consequence of the delay of a few patients, as well as the variability from 0 to 100% in the API in Figure 6. Regarding URG D, only 35 patients (1.4%) were assessed in the lowest urgency category. The evidence of higher cross sectional mean waiting times for those patients was associated to patient initiated admission postponements, due to personal reasons unrelated to the cause of their presence in the list (i.e. other interfering therapies or illnesses). The occurrence of this phenomenon was facilitated by the long MTBT (one year) and by the possibility to delay treatment for patients who were in the least urgent conditions.

As it has happened in Victoria since 2005 [35], proper rules for those cases should be included in the standard waiting list management policy. Our results would likely be enhanced by a more rigorous application of the prioritization algorithm, under a proper admission policy, and with the social/political acknowledgement of the process as a standard rule within the hospital.

### Further validations

The SWALIS model might represent a suitable test bed for different urgency criteria, more objective definitions (i.e. oriented to specific procedures), higher standards (i.e. International Classification of Functioning, Disability and Health), and rater reliability of the clinical assessment. The model might be tested in wider and more heterogeneous environments and its user pool might be expanded, including the management level. Given the model is applied by a complex technological interface, the perceived usefulness and applicability of the prioritization should be assessed together with the user ware of its software environment [36]. The assessment of its clinical impact could include the study of pre-admission and outcome variables, both in time series and in case-control studies.

Since 2003 the SWALIS model has undergone testing in two hospitals in Northern Italy, by different software prototypes (overall 8500 registered patients). In 2007, the Administration of Liguria Region stated the progressive diffusion of the model in its territory and, as of 2009, another general public Hospital in Genoa (counting 6000 surgical admissions per year) will be adopting the model.

## Conclusion

The methods to measure and control WLES are far from being standardized worldwide. The model we propose involves a relevant organizational change in the surgical pre-admission process but it is applicable and appropriate to include different clinical urgency criteria. The SWALIS model would benefit from further validation but according to our experience it allows the use of a homogeneous and standardized method of prioritization. It is suitable for being applied to all patients who are candidates for elective surgery and it allows effective monitoring of waiting lists. As a result, it allows scheduling hospital pre-admission paths efficiently, gathering, retrieving, and sharing considerable information, integrating the different roles in a common environment, and making individuals able to manage problems that need a collaborative solution. It allows orienting resources towards patients in most need, taking a step towards transparency and efficiency, and protecting patients from horizontal and vertical inequities. Due to the accessibility and the applicability of its prioritization algorithm, our model might represent a pragmatic approach to the management of WLES. Its embedding into software environments might be useful in clinical practice and strategic management.

## Abbreviations

API: Appropriate Performance Index; ICD: International Classification of Diseases; MTBT: Maximum Time Before Treatment; OMWT: Overall Mean Waiting Time; NAWDs: Need Adjusted Waiting Days; URGs: Urgency Related Groups; SWALIS: Surgical Waiting List Info System; WLES: Waiting List for Elective Surgery; WP: Waiting Patients.

## Competing interests

The authors declare that they have no competing interests.

## Authors' contributions

RV conceived and coordinated the study, participated in its design, analysis and interpretation of the data, and drafted and supervised the manuscript. AT participated in the study's conception and design, supervised the analysis and interpretation of the data, and co-drafted and revised the manuscript. ET contributed to the study's conception and design, performed analysis and interpretation of the data, and participated in drafting and revising the manuscript. MF participated in the study's conception and design, supervised system development and revised the manuscript. IP contributed to the study's conception and design, performed system development, performed the data management of the study, and participated in drafting and revising the manuscript. MS participated in the study's conception, design and management, in data acquisition and system testing, and participated in revising the manuscript. GS was involved in data analysis, in drafting and revising the manuscript, and revising it critically for important intellectual content. GT participated in the study supervising the system development and testing in the surgical department and gave important intellectual content. GA participated in the study's conception, design and management, in data acquisition and system testing in the surgical department, and participated in revising the manuscript.

All authors read and approved the final manuscript.

## Pre-publication history

The pre-publication history for this paper can be accessed here:


